# Optimal Power Allocation Strategy in a Joint Bistatic Radar and Communication System Based on Low Probability of Intercept

**DOI:** 10.3390/s17122731

**Published:** 2017-11-25

**Authors:** Chenguang Shi, Fei Wang, Sana Salous, Jianjiang Zhou

**Affiliations:** 1 Key Laboratory of Radar Imaging and Microwave Photonics, Ministry of Education, Nanjing University of Aeronautics and Astronautics, Nanjing 211106, China; scg_space@163.com (C.S.); zjjee@nuaa.edu.cn (J.Z.); 2 School of Engineering and Computing Sciences, Durham University, Durham DH1 3DE, UK; sana.salous@durham.ac.uk

**Keywords:** low probability of intercept (LPI), optimal power allocation, joint bistatic radar and communication system, generalized likelihood ratio test (GLRT) detector, probability of detection, probability of false alarm

## Abstract

In this paper, we investigate a low probability of intercept (LPI)-based optimal power allocation strategy for a joint bistatic radar and communication system, which is composed of a dedicated transmitter, a radar receiver, and a communication receiver. The joint system is capable of fulfilling the requirements of both radar and communications simultaneously. First, assuming that the signal-to-noise ratio (SNR) corresponding to the target surveillance path is much weaker than that corresponding to the line of sight path at radar receiver, the analytically closed-form expression for the probability of false alarm is calculated, whereas the closed-form expression for the probability of detection is not analytically tractable and is approximated due to the fact that the received signals are not zero-mean Gaussian under target presence hypothesis. Then, an LPI-based optimal power allocation strategy is presented to minimize the total transmission power for information signal and radar waveform, which is constrained by a specified information rate for the communication receiver and the desired probabilities of detection and false alarm for the radar receiver. The well-known bisection search method is employed to solve the resulting constrained optimization problem. Finally, numerical simulations are provided to reveal the effects of several system parameters on the power allocation results. It is also demonstrated that the LPI performance of the joint bistatic radar and communication system can be markedly improved by utilizing the proposed scheme.

## 1. Introduction

### 1.1. Background and Motivation

Traditionally, a typical radar system is utilized to detect and track targets, whereas the primary objective of a communication system is to transfer information from a source to a sink and then recover that information reliably [1]. These two systems operate in different frequency bands such that they do not interfere with each other. However, due to services with high bandwidth requirements and an exponential increase in the number of wireless devices, the radio frequency (RF) spectrum congestion has become an essential problem. As such, various schemes such as power control, waveform optimization, dynamic spectrum sensing and management can be employed by either radar or wireless communication systems for spectrum sharing [2,3,4]. In recent years, the joint design of radar and communications systems has been regarded as a promising solution to replace traditional spectrum access methods, which is a primary investigation and put forward as a challenging topic at both theoretical and implementation stages [5,6].

Previously, extensive efforts have been made to design an integrated radar and communication system. In [7], a novel waveform design method for joint radar-communication systems based on the Fractional Fourier Transform (FrFT) was presented, where the FrFT was utilized to embed data into chirp sub-carriers with different time-frequency rates. Scharrenbroich and Zatman developed a joint radar-communication systems resource management framework [8], and it was shown that the synergistic spectrum sharing system can provide more radar and communications capacity than either a stand-alone radar system or a stand-alone communications system. The problem of parameter estimation in an OFDM based joint wideband radar and communication system was investigated in [9], and an interpolation based coherent multidimensional parameter estimation framework was proposed. It was also demonstrated that the presented method can significantly outperform the direct application of a multidimensional parameter estimation algorithm to the wideband model. In [10], the authors studied the joint design technique of transmitting sequences and receiving filters subject to peak-to-average ratio constraint in radar and communications systems. The authors in [11,12,13] considered a joint radar and communications system, where several mutual information (MI)-based radar waveform design criteria were developed for spectrum sharing. The numerical results demonstrated that the radar detection performance can be improved by exploiting the scattering due to communication signals at the radar receiver. More recently, Bica et al. investigated the problem of time delay estimation for coexisting multicarrier radar and communications systems [14]. It was also shown that a radar can improve its target estimation performance by utilizing the communication signals scattered off the target in a passive way. In [15], Zhang et al. presented a quasi-orthogonal multi-carrier waveform optimization approach for the joint radar and communication system, and it was shown that the proposed waveform can meet the requirement of the target detection and multi-user transmission at the same time, which is suitable for intelligent transportation systems. Chalise et al. analyzed the performance trade-off for a unified passive radar and communication system [1], and the probabilities of false alarm and detection were computed. In [16], an integrated radar and communication system based on MIMO orthogonal frequency division multiplexing (OFDM) waveform was proposed, and the system parameters, such as the number of subcarriers, subcarrier spacing, and length of cyclic prefix were optimized to satisfy the basic requirements of both radar and communication systems. With the rapid development of advanced driver-assisted systems, an adaptive IEEE 802.11ad waveform was optimally designed for a joint automotive communication and radar system by varying the preamble duration [17], in which a rate distortion-based minimum mean-square error (MMSE) was used as a metric for the trade-off between radar parameters’ estimation accuracy and communication rate. It was indicated that the obtained results can be extended to a large number of joint radar and communication frameworks. Moreover, a novel radar-embedded communication framework is proposed in [18], which is based on the remodulation of the incident radar signaling. Later, the authors in [19,20] develop a multi-objective, optimization paradigm-based waveform design procedure, where the symbol error rate and the intercept metric of the designed waveform are assessed.

### 1.2. Brief Literature Survey of Low Probability of Intercept (LPI) Optimization

Since the notion of LPI design has been an essential and topical part of military operations in modern electronic warfare, LPI performance optimization is a primary issue that needs to be taken into account in designing radar systems. The definition of an LPI radar is a radar system that employs a special emitted waveform intended to prevent a non-cooperative intercept receiver from intercepting, detecting, classifying and identifying its emission. It is necessary to dynamically schedule the radar resources to minimize the probability of intercept for a specified system performance requirement. Technically speaking, low transmit power, short dwell time (time on target), a large revisit interval, and waveform agility will lead to better LPI performance. Thus, many algorithms have been extensively developed within the radar research community to tackle some issues and to improve LPI performance for radar systems, and some of the noteworthy works include [21,22,23,24,25,26,27,28,29,30,31,32,33,34]. To be specific, in [21], Stove et al. investigated the relationship between advanced LPI radar designs and future trends in electronic surveillance measures (ESM) receiving capability, and the key factors influencing the detectability of LPI radar systems were analyzed. In [22], Krishnamurtry presented the efficient dynamic emission management algorithms for multiple networked platforms, which was formulated as a partially observed Markov decision process (POMDP). Fancey and Alabaster proposed a input parameters-based metrication of LPI performance [23], and it was demonstrated that the formula can be utilized to rank waveforms for the purpose of LPI system optimization. In [24], the low probability of exploitation (LPE) performance of LPI radar signal was evaluated by employing the Neural Networks approach, and the simulation results showed that Poly-Phase Shift Keying signal has the best LPE performance. The LPI optimization strategies for distributed radar networks were investigated in [25,26,27,28,29,30,31], where it was demonstrated that radar networks with widely separated transmitters and receivers can provide remarkable LPI performance advantages over traditional monostatic radar systems due to the increased geometric and signal diversities. In [25], a novel LPI-based resource management for target tracking in radar networks was developed, in which the LPI performance was considerably improved by optimizing the revisit interval, dwell time, and transmit power with time difference of arrival (TDOA) cooperation. The work in [26,27,28] focused on the problem of joint target assignment and power allocation in a multi-target environment for LPI radar networks, such that the intercept probability of a radar network was minimized. Recently, the resource scheduling scheme of a radar network system for target tracking in clutter was studied in [29,30], where the sampling interval, transmit power, and waveform parameters were selected for better LPI performance and target tracking accuracy. Game theory provides an efficient mathematical tool to analyze the cooperation and conflict between rational and selfish players. In [31], a cooperative Nash bargaining (NB) power allocation game was formulated for radar networks to minimize the total transmit power. The proof of the existence and uniqueness of the NB solution were presented analytically, which converged quickly to a Pareto optimal equilibrium for the cooperative game.

Moreover, with the exact knowledge of the communication signals, the target spectra and the propagation losses of corresponding channels, the LPI-based adaptive radar waveform optimization algorithms in signal-dependent clutter for joint radar and cellular communication systems were presented for the first time [32], where the communication signals scattered off the target were considered as useful energy, as interference or ignored altogether. The SINR was employed as a metric for target detection, and the signal dependent clutter was considered in the radar waveform design. Nevertheless, the perfect target spectra are usually unavailable because the exact target-radar orientation is practically imprecise, whereas the aforementioned algorithm in [32] assumed that the precise target spectra were available, which were no longer valid in the presence of target spectra uncertainties. Thus, the reference [33] presented several power minimization-based robust radar waveform design criteria to minimize the worst-case radar transmitted power by optimizing the OFDM radar waveform, where the target spectra were assumed to lie in uncertainty sets bounded by known upper and lower bounds. In [34], the problem of LPI performance-based OFDM radar jamming power allocation was studied for the joint radar and communication system, whose purpose was to minimize the total transmitted jamming power by optimizing the multicarrier jamming power allocation while the achieved MI between the received echoes and the target impulse response was enforced to be less than a specified threshold. Overall speaking, the previous literatures lay a solid foundation for the LPI optimization in different radar systems, and it is worthwhile to note that the LPI performance of radar systems can be enhanced by optimizing the revisit interval, transmission power, dwell time, and transmitted waveform while satisfying a given system performance. However, to the best of our knowledge, the problem of LPI-based optimal power allocation in a joint bistatic radar and communication system has not been fully addressed until now.

### 1.3. Main Contributions

In this paper, motivated by the results in [1,35], we investigate the LPI-based optimal power allocation strategy for a joint bistatic radar and communication system, where the joint system consists of a dedicated transmitter, a radar receiver, and a communication receiver. The joint system is capable of fulfilling the requirements of both radar and communications simultaneously. It is worth pointing out that the proposed optimal power allocation strategy is particularly attractive for target tracking in which the location and velocity of the target are perfectly estimated, but fine detection performance is required to retrieve the exact target location and characteristics. In this scenario, the primary objective of the joint system is to secure a predetermined information rate constraint for the communication receiver and specified probabilities of false alarm and detection for the radar receiver, while minimizing the total transmission power for both the information signal and radar waveform in the joint system. It should be noted that the dedicated transmitter can minimize the transmission power during the dwell time to achieve LPI performance while extending the integration time to maintain the radar receiver’s sensitivity.

The major contributions of this work are listed as follows:

(1) We formulate the system model and derive the generalized likelihood ratio test (GLRT) detector for a joint bistatic radar and communication system, which is composed of a dedicated transmitter, a radar receiver, and a communication receiver. The dedicated transmitter emits a portion of the total transmission power to broadcast information signal, and the other portion is employed for transmitting a radar waveform. The goal of the transmitter is to detect enemy targets as well as to transfer information to a communication receiver, which then recovers that information reliably [1]. This is quite different from the dual function system [18], which can only transmit the same signal for both radar and communication functions.
(2) Assuming that the signal-to-noise ratio (SNR) corresponding to the target surveillance path is much weaker than that corresponding to the direct path at the radar receiver, the analytically closed-form expression for the probability of false alarm is derived, whereas the closed-form expression for the probability of detection is not analytically tractable and is approximated due to the fact that the received signals are not zero-mean Gaussian under target presence hypothesis.
(3) The problem of LPI-based optimal power allocation in a joint bistatic radar and communication system is investigated, which minimizes the total transmission power for both information signal and radar waveform while satisfying a specified information rate for communication receiver and desired probabilities of detection and false alarm for radar receiver.
(4) The proposed LPI-based optimal power allocation strategy is solved numerically, and the bisection search technique is exploited to find the optimal solution for the aforementioned optimization problem. It is shown that significant computational savings can be obtained through the utilization of bisection method when compared with the exhaustive search approach.
(5) Numerical simulation results demonstrate the superiority of the proposed LPI-based optimal power allocation scheme in terms of the LPI performance of the joint system. Moreover, precious results in [1] address the performance trade-off between the radar and communication subsystems, which only analyze the boundaries of the probability of false alarm-information rate and the probability of detection-information rate regions. As an extension, the effects of total transmit power, probability of false alarm, SNRs of different paths, and communication rate on the probability of detection are discussed in this study. To be specific, it can be concluded via simulations that the probability of detection is a function of transmit power, probability of false alarm, and SNRs of different paths, as well as the information rate for communication receiver.

### 1.4. Outline of the Paper

The remainder of this paper is structured as follows. The considered joint bistatic radar and communication system model as well as the underlying assumptions needed in this paper are introduced in Section 2. In Section 3, the LPI-based optimal power allocation strategy is developed. In Section 3.1, the basis of the power allocation strategy is introduced. Section 2.2 analyzes the GLRT detector. The resulting constrained optimization problem is solved by the well-known bisection search method in Section 3.3, followed by discussion in Section 3.4. Several numerical simulations are provided in Section 4 to verify the accuracy of the theoretical calculations as well as demonstrate the effectiveness of the proposed LPI-based power allocation scheme. Finally, the concluding remarks of this paper are summarized in Section 5.

**Notations:** Bold lower-case letters and bold upper-case letters are utilized for column vectors and matrices, respectively. ${\left(\cdot \right)}^{H}$ denotes Hermitian transpose, I denotes the identity matrix, and ∥·∥ stands for the Euclidean norm. Pr{·} is the probability operator. $\mathrm{CN}(\mu ,\sigma^{2})$ denotes Gaussian distribution with mean μ and variance $\sigma^{2}$. Γ(x) represents the Gamma function, and $Q_{1}\left(a,b\right)$ represents the first-order Marcum Q-function with parameters *a* and *b*.

## 2. System and Signal Models

### 2.1. Problem Scenario

**Figure 1:**
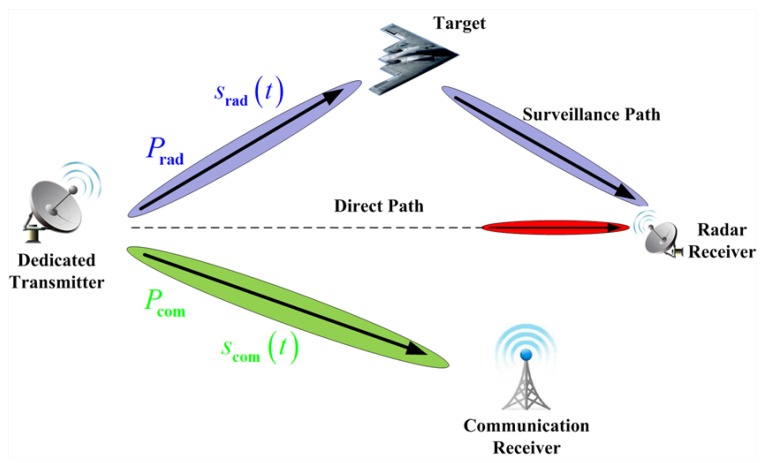
Joint bistatic radar and communication system with a dedicated transmitter, a radar receiver, and a communication receiver.

Let us consider a joint bistatic radar and communication system that consists of a dedicated transmitter, a radar receiver and a communication receiver. Such a joint system with a single point target is depicted in Figure 1. The objective of the dedicated transmitter is to detect and track enemy targets, as well as to transfer information to a communication receiver, which can recover that information reliably [1]. The dedicated transmitter employs a portion of the total transmission power to broadcast a radar waveform $s_{\mathrm{rad}}\left(t\right)$, whereas the other portion is utilized for broadcasting information signal $s_{\mathrm{com}}\left(t\right)$, where $P_{\mathrm{rad}}$ and $P_{\mathrm{com}}$ denote the transmission power allocated for the radar and information waveforms respectively. Without loss of generality and to simplify the analysis, it is assumed that the radar and communication signal transmissions are optimally scheduled by employing non-overlapping groups of resource (time-frequency) element units. To be specific, some element units are used as a radar transmitter, while the other units are utilized as a communication transmitter, which work in different frequency bands for their corresponding operations such that they do not interfere with each other. Hence, the mutual interference received by the radar receiver and communication receiver can be minimized.

The radar receiver works with an antenna directed to the dedicated transmitter to receive the direct path (dedicated transmitter to radar receiver) signal, and another one illuminates the target to receive the scattered echoes. It is supposed that the radar receiver is capable of adaptive beamforming in two paths, one required for target surveillance and one for receiving the reference radar signal through the direct path [14]. Adaptive beamfoming is able to reject interferences from other angles. Moreover, the successive interference cancellation (SIC) technique is utilized at the radar receiver to remove the strong line of sight radar signal from the observed signal, where the interference-free radar return can be obtained. For instance, the time difference for the received signals at the radar receiver between the two paths can be calculated by employing a correlation between the reference signal and target surveillance signal. From this, the reconstructed reference signal can be removed from the target surveillance signal and the interference free radar signal is obtained.

**Remark** **1.** *It is well known that the bistatic geometry plays an important role in the ambiguity function [36]. Specifically, the effects of geometry factors become more and more prominent in the regions close to the baseline, whereas the resolution is totally lost when the target is on the baseline. For simplicity, it is assumed that the target is far from the baseline. The LPI-based optimal power allocation taking into account the geometry factors will be investigated in the future*.

**Remark** **2.** *The crucial problem associated with bistatic radar system is the time and frequency synchronization at the transmitter and receiver for coherent signal processing and target detection [37]. Time and frequency synchronization can be easily obtained by employing modern communications satellites and global positioning system (GPS) signals, which can provide a highly stable pulse-per-second (PPS) signal. Utilizing the PPS signal to synchronize a stable quartz crystal oscillator, a standard derivation of less than 5 ns is achieved. In addition, frequency synchronization can be realized by generating all needed frequencies by dividing, multiplying or phase-locking to the GPS disciplined oscillators at the transmitter and receiver*.

At the communication receiver, the information rate for the communication system in bits per channel use (bpcu) can be expressed as:

(1) $$\begin{array}{c} R=\log_{2}\left(1+P_{\mathrm{com}}\gamma_{\mathrm{com}}\right), \end{array}$$

where $\gamma_{\mathrm{com}}$ denotes the ratio of the squared absolute value of the dedicated transmitter–communication receiver path to the variance of additive noise plus the transmitted signal scattered off the target at the communication receiver. As such, $P_{\mathrm{com}}\gamma_{\mathrm{com}}$ stands for the instantaneous SNR at the communication receiver. It should be noted that we will concentrate on the joint bistatic radar and communication system with a dedicated transmitter, a radar receiver and a communication receiver in the rest of this paper. In the case of multiple-input multiple-output (MIMO) systems, the analysis is much more complex. In future work, the model and derivations will be extended to the MIMO system scenario.

### 2.2. Signal Model

In this paper, it is assumed that there is a single point target. Then, the target detection problem can be turned to a binary hypothesis testing problem, with $H_{1}$ corresponding to the target presence hypothesis and $H_{0}$ corresponding to the null hypothesis. Therefore, the *K* time-domain samples of the received signals for radar receiver can be expressed as the complex K×1 vectors [35,38]:

(2) $$H_{0}\;(\mathrm{no}\;\mathrm{target}\;\mathrm{present}):\left\{\begin{array}{cc} r_{d} & =\gamma_{d}U_{d}s_{\mathrm{rad}}+n_{d}\\ r_{t} & =n_{t}, \end{array}\right.$$

(3) $$H_{1}\;(\mathrm{target}\;\mathrm{present}):\left\{\begin{array}{cc} r_{d} & =\gamma_{d}U_{d}s_{\mathrm{rad}}+n_{d}\\ r_{t} & =\gamma_{t}U_{t}s_{\mathrm{rad}}+n_{t}, \end{array}\right.$$

where $r_{d}$ and $r_{t}$ denote the received signals corresponding to the reference and surveillance paths respectively, $s_{\mathrm{rad}}$ denotes the K×1 vector of sampled radar waveform, whose transmission power is $P_{\mathrm{rad}}$, $\gamma_{d}$ and $\gamma_{t}$ denote the scalar attenuation loss coefficients corresponding to the reference path and surveillance path respectively. $U_{d}$ and $U_{t}$ represent the K×K unitary delay-Doppler operator matrices corresponding to the two paths, $n_{d}$ and $n_{d}$ are the radar receiver noise at the antennas used for the reference and surveillance paths, respectively.

Generally speaking, the parameters $\gamma_{d}$ and $\gamma_{t}$ are unknown. Since the position of the dedicated transmitter and the position and velocity of the moving target at a range-Doppler cell (hypothesized position) are known, $U_{d}$ and $U_{t}$ can be calculated. Furthermore, due to the fact that $U_{d}$ and $U_{t}$ are unitary matrices, that is, $U_{d}U_{d}^{H}=U_{t}U_{t}^{H}=I$, the received signals in (2) and (3) can be simplified by employing unitary transformations as:

(4) $$H_{0}\;(\mathrm{no}\;\mathrm{target}\;\mathrm{present}):\left\{\begin{array}{cc} \tilde{r_{d}} & =\gamma_{d}s_{\mathrm{rad}}+\tilde{n_{d}}\\ \tilde{r_{t}} & =\tilde{n_{t}}, \end{array}\right.$$

(5) $$H_{1}\;(\mathrm{target}\;\mathrm{present}):\left\{\begin{array}{cc} \tilde{r_{d}} & =\gamma_{d}s_{\mathrm{rad}}+\tilde{n_{d}}\\ \tilde{r_{t}} & =\gamma_{t}s_{\mathrm{rad}}+\tilde{n_{t}}, \end{array}\right.$$

where $\tilde{r_{d}}=U_{d}^{H}r_{d}$, $\tilde{r_{t}}=U_{t}^{H}r_{t}$. $\tilde{n_{d}}=U_{d}^{H}n_{d}$, $\tilde{n_{t}}=U_{t}^{H}n_{t}$, which denote the additive zero-mean white Gaussian noise with variance matrix $\sigma_{n}^{2}I$, that is, $\tilde{n_{d}}∼\mathrm{CN}\left(0,\sigma_{n}^{2}I\right)$, $\tilde{n_{t}}∼\mathrm{CN}\left(0,\sigma_{n}^{2}I\right)$. In the following, an exact expression for probability of false alarm and an approximated expression for probability of detection will be derived.

**Remark** **3.** *Without loss of generality, we concentrate on a single-target scenario in this study. However, the derivations and results can be extended to the multiple-target case, where the dedicated transmitter can launch multiple beams simultaneously to detect several targets independently [39]. Each beam can be utilized to detect one target, and thus, multiple targets can be illuminated in this working mode. It is worth mentioning that the consumption of the transmission power in the multibeam technique grows significantly with the number of targets. Hence, at any illumination, the total transmission power of the multiple beams needs to be constrained so that the consumption power of the dedicated transmitter cannot surpass the endurable ability of system physical equipment*.

## 3. Problem Formulation

### 3.1. Basic of the Technique

Mathematically, the LPI-based optimal power allocation strategy can be formulated as a problem of optimizing the transmit power to minimize the total transmission power for both information signal and radar waveform subject to some system constraints. Firstly, the GLRT detector is analyzed, where the analytically closed-form expression for the probability of false alarm is calculated, and the closed-form expression for the probability of detection is approximated due to the fact that the received signals are not zero-mean Gaussian under the target presence hypothesis. We are then in a position to optimize and allocate the transmit power for both information signal and radar waveform in order to achieve better LPI performance for the joint system. The general LPI-based optimal power allocation strategy is detailed as follows.

### 3.2. GLRT Detector

In order to design an efficient detector for the hypothesis testing, the GLRT detector is employed here by exploiting the signal model described in the previous section, which is similar to the Neyman-Pearson detector as the number of samples approaches infinity [35]. Based on the above definitions, the joint probability density functions (PDFs) of $\tilde{r_{d}}$ and $\tilde{r_{t}}$ under the hypotheses $H_{0}$ and $H_{1}$ respectively, can be expressed as:

(6) $$\begin{array}{c} f\left(\left(\tilde{r_{d}},\tilde{r_{t}}\right)|H_{0}\right)=\frac{1}{{\left(\pi \sigma_{n}^{2}\right)}^{2K}}\exp\left(\frac{-∥\tilde{r_{d}}-\gamma_{d}s_{\mathrm{rad}}{∥}^{2}-{∥\tilde{r_{t}}∥}^{2}}{\sigma_{n}^{2}}\right), \end{array}$$

and

(7) $$\begin{array}{c} f\left(\left(\tilde{r_{d}},\tilde{r_{t}}\right)|H_{1}\right)=\frac{1}{{\left(\pi \sigma_{n}^{2}\right)}^{2K}}\exp\left(\frac{-∥\tilde{r_{d}}-\gamma_{d}s_{\mathrm{rad}}{∥}^{2}-{∥\tilde{r_{t}}-\gamma_{t}s_{\mathrm{rad}}∥}^{2}}{\sigma_{n}^{2}}\right). \end{array}$$

Let $\bar{\lambda }\in \left[0,1\right]$ represents a certain detection threshold for the hypothesis testing for radar receiver. Then, the target detection problem can be formulated and solved by comparing the GLRT function as:

(8) $$\Upsilon \left(\tilde{r_{d}},\tilde{r_{t}}\right)=\frac{f(\left(\tilde{r_{d}},\tilde{r_{t}}\right)|H_{1})}{f(\left(\tilde{r_{d}},\tilde{r_{t}}\right)|H_{0})}=\exp\left(\frac{∥\tilde{r_{t}}{∥}^{2}-{∥\tilde{r_{t}}-\gamma_{t}s_{\mathrm{rad}}∥}^{2}}{\sigma_{n}^{2}}\right)\overset{H_{1}}{\underset{H_{0}}{≷}}\bar{\lambda },$$

where the threshold for hypothesis testing $\bar{\lambda }$ can be determined by the predefined probability of false alarm $p_{\mathrm{FA}}$. As aforementioned, since the parameters $\gamma_{d}$ and $\gamma_{t}$ are not known, they are substituted with their estimated values in the GLRT function. Note that the main contribution of this work lies in the power minimization based optimal power allocation strategy described above. The different target detector models [40,41,42] are not compared because they are out of the scope of this paper.

With the derivations in [1,38], the probability of false alarm $p_{\mathrm{FA}}$ can be given by:

(9) $$\begin{array}{c} p_{\mathrm{FA}}=\exp\left(-\lambda \right)+\frac{2\lambda \exp\left[-(\lambda +\frac{\beta }{2})\right]}{2^{K}\Gamma \left(K\right)}\left\{\sum_{m=0}^{K-2}\sum_{n=0}^{m}\frac{m!}{n!}{\left(2\lambda \right)}^{m-n}I_{1}-\sum_{m=0}^{K-2}\sum_{p=0}^{m}\frac{m!}{n!}\sum_{n=0}^{m}\frac{1}{p!2^{p}}{\left(2\lambda \right)}^{m-n}I_{2}\right\}, \end{array}$$

where $\lambda =\ln(\bar{\lambda })$, $\beta =\frac{2|\gamma_{d}^{2}|P_{\mathrm{rad}}}{\sigma_{n}^{2}}$, and

(10) $$\begin{array}{c} I_{1}=\int_{0}^{+\infty }y^{(K+n-m-1)-1}e^{-\frac{y}{2}}{}_{0}F_{1}\left(;K;\frac{\beta }{4}y\right)dy, \end{array}$$

(11) $$\begin{array}{c} I_{2}=\int_{0}^{+\infty }y^{(K+k+n-m-1)-1}e^{-\frac{y}{2}}{}_{0}F_{1}\left(;K;\frac{\beta }{4}y\right)dy, \end{array}$$

where ${}_{0}F_{1}\left(;a;x\right)$ denotes the hypergeometric function as shown in [43]. As computed in [1], Equation (9) can be rewritten as:

(12) $$\begin{array}{cc} p_{\mathrm{FA}} & =\exp\left(-\lambda \right)+\frac{2\lambda \exp\left[-(\lambda +\frac{\beta }{2})\right]}{2^{K}\Gamma \left(K\right)}\left\{\sum_{m=0}^{K-2}\sum_{n=0}^{m}\frac{m!}{n!}2^{K-1}\lambda^{m-n}\Gamma \left(K+n-m-1\right){}_{1}F_{1}\left(K+n-m-1;K;\frac{\beta }{2}\right)\right.\\ & \left.-\sum_{m=0}^{K-2}\sum_{p=0}^{m}\sum_{n=0}^{m}\frac{m!}{n!}\frac{2^{m-n-p}\lambda^{m-n}}{p!}\Gamma \left(K+1+n-m-1\right){}_{1}F_{1}\left(K+p+n-m-1;K;\frac{\beta }{4}\right)\right\}, \end{array}$$

where ${}_{1}F_{1}\left(a;b;x\right)$ is the hypergeometric function [36]. In this paper, it is assumed that the SNR corresponding to the reference path is much larger than that corresponding to the surveillance path, i.e., $\gamma_{\mathrm{ref}}=\frac{|\gamma_{d}{|}^{2}}{\sigma_{n}^{2}}$ has large value. Consequently, Equation (12) can be approximated as follows:

(13) $$\begin{array}{c} p_{\mathrm{FA}}≃\exp\left(-\lambda \right). \end{array}$$

where the threshold value λ can be computed from the specified probability of false alarm $p_{\mathrm{FA}}$.

As for the probability of detection $p_{D}$, the closed-form expression for $p_{D}$ is not obtainable. This is because $\tilde{r_{t}}|H_{1}∼\mathrm{CN}\left(\gamma_{t}s_{\mathrm{rad}},\sigma_{n}^{2}I\right)$, which is not zero-mean Gaussian under the hypothesis $H_{1}$. In the case of high $\gamma_{\mathrm{ref}}$ value, the probability of detection $p_{D}$ can be approximately derived as:

(14) $$\begin{array}{cc} p_{D} & ≃\mathrm{Pr}\left\{\frac{2}{\sigma_{n}^{2}}{\tilde{r_{t}}}^{H}\frac{s_{\mathrm{rad}}s_{\mathrm{rad}}^{H}}{P_{\mathrm{rad}}}\tilde{r_{t}}\geq 2\lambda |H_{0}\right\}\\ & ≃Q_{1}\left(\sqrt{2P_{\mathrm{rad}}\gamma_{\mathrm{rad}}},\sqrt{2\lambda }\right), \end{array}$$

where $\gamma_{\mathrm{rad}}=\frac{|\gamma_{t}{|}^{2}}{\sigma_{n}^{2}}$. Technically speaking, the radar receiver’s performance can be assessed in terms of the probability of detection $p_{D}$ and the probability of false alarm $p_{\mathrm{FA}}$[35]. It can be deduced from (14) that $p_{D}$ is increased monotonically with $P_{\mathrm{rad}}$, meaning better target detection performance. Nevertheless, from a practical point of view, it also leads to transmitting much more power, which would increase the vulnerability of the joint system in modern electronic warfare. Therefore, we formulate our LPI-based power allocation problem by minimizing the total transmit power while guaranteeing given probabilities of detection and false alarm, as proposed in the next subsection.

### 3.3. LPI-Based Optimal Power Allocation Strategy

In this paper, we concentrate on the LPI-based optimal power allocation strategy for a joint bistatic radar and communication system, whose purpose is to minimize the total transmission power for information signal and radar waveform while guaranteeing predetermined probabilities of detection and false alarm for the radar receiver. We impose a minimum information rate constraint per channel for the communication receiver and for the considered system an upper bound on the total transmit power. In such a model, the LPI performance for the joint system can evidently be enhanced. Eventually, the resulting optimal power allocation strategy can be formulated as follows:

(15a) $$\begin{array}{cc} P1:\;\;\; & \min_{P_{\mathrm{rad}},P_{\mathrm{com}}}\;\;P_{\mathrm{Total}}=P_{\mathrm{rad}}+P_{\mathrm{com}}, \end{array}$$

(15b) $$\begin{array}{c} s.t.:\;\left\{\begin{array}{c} C1:R\geq r_{\mathrm{th}},\\ C2:p_{\mathrm{FA}}\leq \delta_{\mathrm{FA}},\\ C3:p_{D}\geq \delta_{D}. \end{array}\right. \end{array}$$

where $P_{\mathrm{Total}}$ represents the total transmission power, $r_{\mathrm{th}}$ denotes the given information rate threshold for the communication receiver, $\delta_{D}$ and $\delta_{\mathrm{FA}}$ are the corresponding threshold values for the probabilities of detection and false alarm respectively. The first constraint C1 implies that the information rate for the communication receiver should be above the threshold $r_{\mathrm{th}}$ to guarantee the communication performance. The second one C2 stands that the probability of false alarm is less than a predefined threshold $\delta_{\mathrm{FA}}$, while the third constraint C3 stands that the achieved probability of detection is greater than a specified threshold $\delta_{D}$ such that the required target detection performance is met. As previously stated, the dedicated transmitter can minimize the transmission power during the dwell time to achieve LPI performance while extending the integration time to maintain the radar receiver’s sensitivity.

In the optimization problem P1, the minimum value of $\left(P_{\mathrm{rad}}+P_{\mathrm{com}}\right)$ can be achieved when the first three inequalities are simultaneously active. Then, substituting (1) into $R\geq r_{\mathrm{th}}$, we can obtain:

(16) $$\begin{array}{c} P_{\mathrm{com}}\geq \frac{1}{\gamma_{\mathrm{com}}}\left(2^{r_{\mathrm{th}}}-1\right). \end{array}$$

Solving Equation (16) to obtain $P_{\mathrm{com}}$ and substituting it into constraint C1, then the power allocation problem P1 for a joint bistatic radar and communication system can be reformulated as follows:

(17a) $$\begin{array}{cc} P2:\;\;\; & \min_{P_{\mathrm{rad}},P_{\mathrm{com}}}\;\;P_{\mathrm{Total}}=P_{\mathrm{rad}}+P_{\mathrm{com}}, \end{array}$$

(17b) $$\begin{array}{c} s.t.:\;\left\{\begin{array}{c} C1:P_{\mathrm{com}}\geq \frac{1}{\gamma_{\mathrm{com}}}\left(2^{r_{\mathrm{th}}}-1\right),\\ C2:p_{\mathrm{FA}}\leq \delta_{\mathrm{FA}},\\ C3:p_{D}\geq \delta_{D}. \end{array}\right. \end{array}$$

From the optimization problem P2, it is evident that the optimal transmission power for the information signal is obtained when the equality in constraint C1 holds. To actually find the optimal value of $P_{\mathrm{rad}}$ that ensures the optimum LPI performance while making sure that the constraints are totally satisfied, we employ the well-known bisection search approach. In the following, the detailed steps of the LPI-based optimal power allocation algorithm are summarized in Algorithm 1, according to which we can iteratively obtain the optimum transmit power for the communication rate $P_{\mathrm{com}}^{*}$ and radar waveform $P_{\mathrm{rad}}^{*}$, respectively.

**Remark** **4.** *Nowadays, the trend for modern radar is not only detecting the target, but also including target classification, target recognition, and target imaging. Thus, for radar imaging applications, high resolution is required, whereas minimizing radar transmission power leads to low resolution imagery of target and loses the essence of target imaging. It is noteworthy that we only concentrate on the target detection performance in terms of probabilities of detection and false alarm in this paper. The primary objective of the transmitter is to guarantee a specified information rate for the communication receiver and predetermined probabilities of detection and false alarm for the radar receiver, while allocating the minimum transmission power to the transmitter [23]. In this way, the desired target detection performance can be satisfied. In the future, we will investigate the problem of LPI based optimal power allocation for target imaging, where the constraint of super resolution will be imposed*.

The iterative procedure is detailed in Algorithm 1. The bisection search algorithm is listed as Algorithm 2. It is worth noting that the proposed optimal power allocation strategy is particularly attractive for target tracking in which the location and velocity of the target are perfectly estimated, but fine detection performance is required to track the exact target location. In such case, the aim of our work is to obtain optimal transmit power allocation to guarantee the communication rate and the probabilities of false alarm and detection. In the next section, some numerical examples are presented to support the mathematical analysis.

**Algorithm 1** LPI-Based Optimal Power Allocation Strategy
1:**Initialization:** $\delta_{\mathrm{FA}}$, $\delta_{D}$, $P_{\mathrm{Total}}$, $r_{\mathrm{th}}$, iterative index n=1;  2:**Loop until** $P_{\mathrm{rad}}$ **converges:**   Calculate $P_{\mathrm{com}}$ by solving (16);   Calculate $P_{\mathrm{rad}}^{\left(n\right)}$ via bisection search in **Algorithm 2**;   Calculate $p_{D}^{\left(n\right)}\leftarrow \left(\sqrt{2P_{\mathrm{rad}}^{\left(n\right)}\gamma_{\mathrm{rad}}},\sqrt{2\lambda }\right)$;3:**End loop**  4:**Update:** Update $P_{\mathrm{com}}^{*}\leftarrow P_{\mathrm{com}}$, $P_{\mathrm{rad}}^{*}\leftarrow P_{\mathrm{rad}}^{\left(n\right)}$.


**Algorithm 2** Bisection Search of $P_{\mathrm{rad}}$
1:**Initialization:** $a=P_{\min}-P_{\mathrm{com}}$, $b=P_{\mathrm{Total}}-P_{\mathrm{com}}$, $c^{\left(n\right)}$, $f\left(x\right)=Q_{1}\left(\sqrt{2\gamma_{\mathrm{rad}}x},\sqrt{2\lambda }\right)$, the tolerance ϵ>0;  2:**Loop until:** $p_{D}^{\left(n\right)}-\delta_{D}\geq \epsilon$
  **if** f(a)≥0 **then**     $P_{\mathrm{rad}}^{\left(n\right)}\leftarrow a$ and stop the iteration;  **else**
     $c^{\left(n\right)}\leftarrow \frac{a+b}{2}$;     **if** $f(c^{\left(n\right)})=0$ **then**
      $P_{\mathrm{rad}}^{\left(n\right)}\leftarrow a$ and stop the iteration;     **if** $f(c^{\left(n\right)})<0$ **then**
      $a\leftarrow c^{\left(n\right)}$;      $c^{\left(n\right)}\leftarrow \frac{a+b}{2}$;     **if** $f(c^{\left(n\right)})>0$ **then**
      $b\leftarrow c^{\left(n\right)}$;      $c^{\left(n\right)}\leftarrow \frac{a+b}{2}$;     **end if**     Set n←n+1;  **end if**  3:**End loop** 


### 3.4. Discussion

(1) *Convergence Analysis:* From Algorithm 1 and Algorithm 2, it is worth pointing out that the optimal transmitting power for both information signal $P_{\mathrm{com}}^{*}$ and radar waveform $P_{\mathrm{rad}}^{*}$ can be obtained by solving problem P2 for a specified communication rate and the desired probabilities of detection and false alarm. At the (n+1)th step, the transmit power $P_{\mathrm{rad}}^{\left(n+1\right)}$ is updated from the optimal solution $P_{\mathrm{rad}}^{\left(n\right)}$, determined through the previous solution. Thus, $P_{\mathrm{rad}}^{\left(n+1\right)}$ is always the feasible solution of the next iteration, and the optimal transmission power for radar waveform $P_{\mathrm{rad}}^{\left(n+1\right)}$ will achieve a value of $p_{D}$, which is greater or equal to that of the previous iteration. This shows that the achieved $p_{D}$ value will monotonically increase at each iteration, such that the gap between the temporal probability of detection and the specified $p_{D}$ threshold is minimized. Hence, Algorithm 1 will converge to the optimal solution through the bisection search approach, which is due to the fact that the achievable $p_{D}$ is upper bounded for a given transmit power for radar waveform.

(2) *Complexity Analysis:* The computational complexity of Algorithm 1 is dominated by the procedure of bisection search method. The convergence rate of Algorithm 1 is based on the bisection search method, which is given by $O(\log_{2}\left[\left(b-a\right)/\epsilon \right])$, while the exhaustive search has a complexity of O((b-a)/ϵ). For example, a system with ϵ=0.1, a=0, b=800, and $P_{\mathrm{rad}}=520$ would require only in the order of 12.9658 iterations with the proposed strategy, whereas the exhaustive search approach requires on the order of 5200 iterations. This indicates that the proposed LPI-based optimal power allocation strategy requires only 0.2493% of the iterations compared with the exhaustive search. It should be highlighted that a significant computational saving can be achieved through the utilization of the presented strategy for great threshold of probability of detection. In addition, the gap goes up rapidly with the increase of $\gamma_{D}$.

## 4. Numerical Simulations and Performance Analysis

### 4.1. Description

In this section, numerical examples are provided to verify the accuracy of the theoretical derivations as well as to demonstrate the improvement of the LPI performance brought by our presented optimal power allocation strategy. For numerical simulations, we assume a joint bistatic radar and communication system composed of a dedicated transmitter, a radar receiver, and a communication receiver. Before the initialization of the proposed strategy, we should first determine the target detection threshold γ. Then, for a given γ, (13) and (14) can be used to decide the desired probabilities of false alarm and detection, i.e., $\delta_{\mathrm{FA}}$ and $\delta_{D}$, respectively. In the considered system, the corresponding target detection threshold γ is set to be 13.8155, and the desired probabilities of false alarm and detection can be obtained as $\delta_{\mathrm{FA}}=10^{-6}$ and $\delta_{D}=0.9$ for the radar receiver. In addition, we set the specified threshold of information rate at $r_{\mathrm{th}}=5\;\mathrm{bpcu}$.

### 4.2. Simulation Results

**Figure 2:**
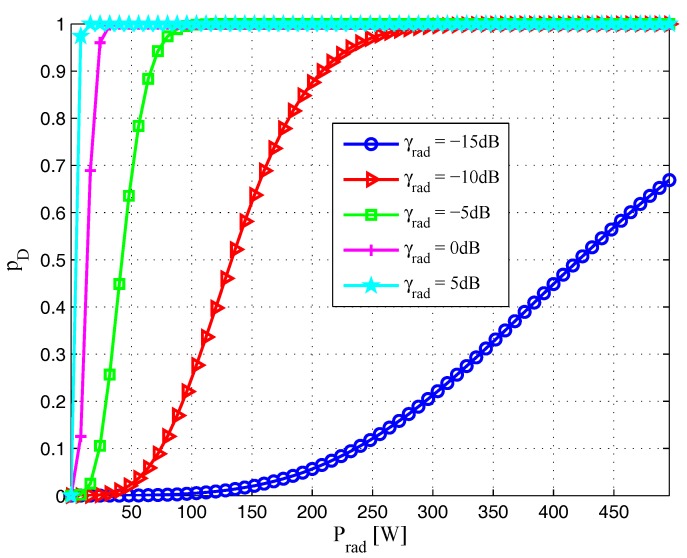
Probability of detection $p_{D}$ versus transmit power for radar waveform $P_{\mathrm{rad}}$ with different $\gamma_{\mathrm{rad}}$ ($\delta_{\mathrm{FA}}=10^{-6}$).

In Figure 2, we plot the probability of detection $p_{D}$ with respect to the transmission power for radar waveform $P_{\mathrm{rad}}$ for different $\gamma_{\mathrm{rad}}$, i.e., $\gamma_{\mathrm{rad}}=-15\;\mathrm{dB}$, $\gamma_{\mathrm{rad}}=-10\;\mathrm{dB}$, $\gamma_{\mathrm{rad}}=-5\;\mathrm{dB}$, $\gamma_{\mathrm{rad}}=0\;\mathrm{dB}$, and $\gamma_{\mathrm{rad}}=5\;\mathrm{dB}$. One can observe that the boundaries of the regions widen with an increase in $\gamma_{\mathrm{rad}}$. That is to say, the probability of detection increases more drastically when $\gamma_{\mathrm{rad}}$ goes up, which implies that it is desirable to have a large value of $\gamma_{\mathrm{rad}}$ to achieve good target detection performance, as expected.

**Figure 3:**
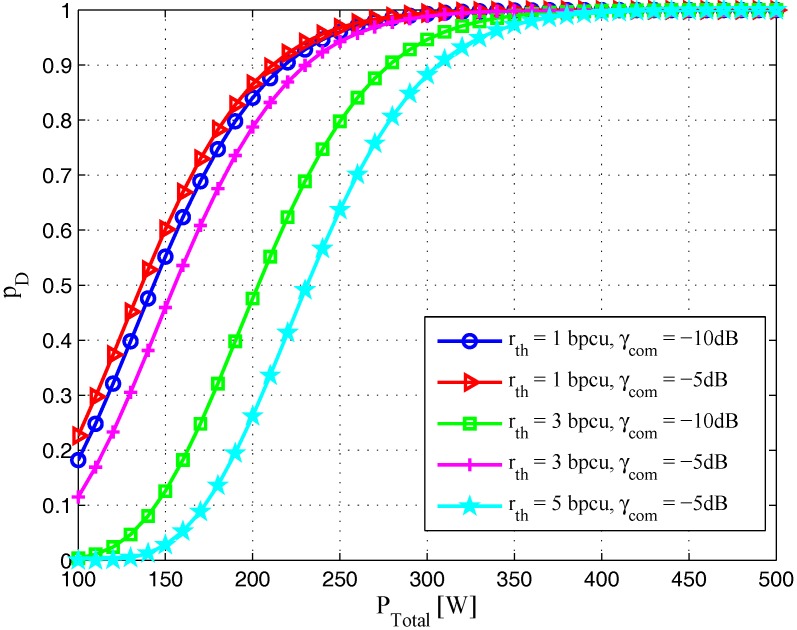
Probability of detection $p_{D}$ versus total transmit power $P_{\mathrm{Total}}$ with different $r_{\mathrm{th}}$ and $\gamma_{\mathrm{com}}$ ($\delta_{\mathrm{FA}}=10^{-6}$, $\gamma_{\mathrm{rad}}=-10\;\mathrm{dB}$).

To get more insights, the effects of some system parameters on target detection performance are discussed via simulations. Figure 3 plots the probability of detection $p_{D}$ versus total transmit power $P_{\mathrm{Total}}$ for different $r_{\mathrm{th}}$ and $\gamma_{\mathrm{com}}$. It is obvious that the probability of detection is increased when the total transmit power of the joint system $P_{\mathrm{Total}}$ increases. On the other hand, with the increase of $r_{\mathrm{th}}$ and decrease of $\gamma_{\mathrm{com}}$, the boundaries of the regions widen as well. This is due to the fact that the available transmission power for radar waveform is reduced when $r_{\mathrm{th}}$ increases and $\gamma_{\mathrm{com}}$ decreases, which results in a lower probability of detection and worse target detection performance.

**Figure 4:**
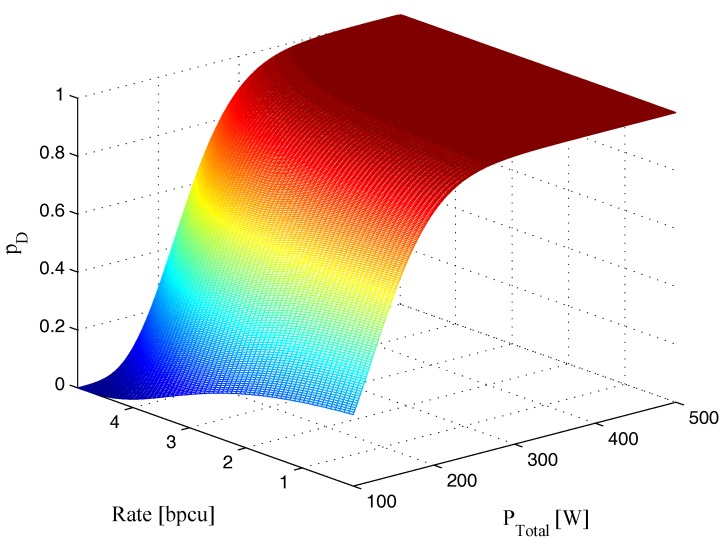
Variation in probability of detection $p_{D}$ with change in total transmit power $P_{\mathrm{Total}}$ and information rate *R* ($\delta_{\mathrm{FA}}=10^{-6}$, $\gamma_{\mathrm{rad}}=-10\;\mathrm{dB}$, $\gamma_{\mathrm{com}}=-5\;\mathrm{dB}$).

**Figure 5:**
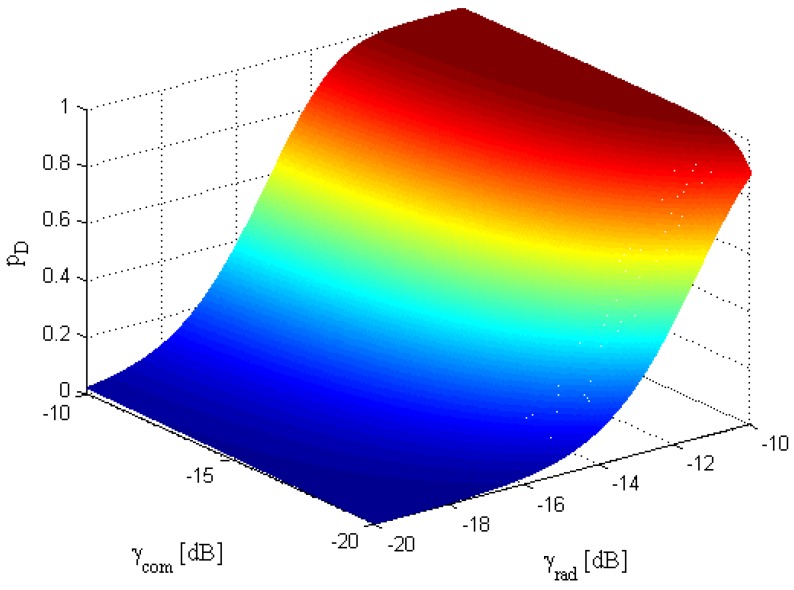
Variation in probability of detection $p_{D}$ with change in $\gamma_{\mathrm{rad}}$ and $\gamma_{\mathrm{com}}$ ($P_{\mathrm{rad}}=500\;W$, $\delta_{\mathrm{FA}}=10^{-6}$, $r_{\mathrm{th}}=2\;\mathrm{bpcu}$).

Additionally, Figure 4 shows the variation in $p_{D}$ with change in $P_{\mathrm{Total}}$ and *R*. We can clearly observe from Figure 4 that the probability of detection for the radar receiver is gradually increased and achieves the maximum value with a decrease in $r_{\mathrm{th}}$ and an increase in $\gamma_{\mathrm{com}}$. In Figure 5, the effect of $\gamma_{\mathrm{rad}}$ and $\gamma_{\mathrm{com}}$ on the probability of detection $p_{D}$ is illustrated, which verifies that the probability of detection increases when $\gamma_{\mathrm{rad}}$ and $\gamma_{\mathrm{com}}$ increase. Hence, it can be concluded from these figures that the probability of detection is a function of transmit power, probability of false alarm, SNRs of different paths, as well as information rate for communication receiver.

**Figure 6:**
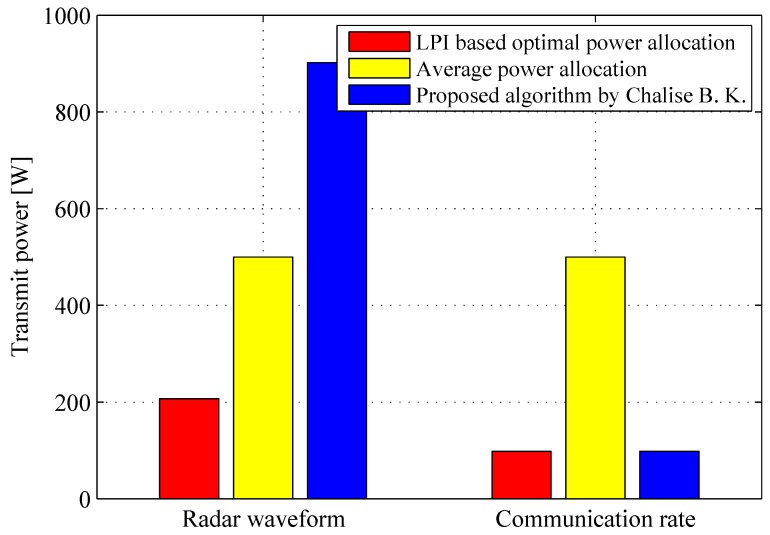
Comparisons of transmit power for radar waveform and communication rate employing different algorithms ($P_{\mathrm{Total}}=1000\;W$, $\delta_{D}=0.9$, $\delta_{\mathrm{FA}}=10^{-6}$, $\gamma_{\mathrm{rad}}=-10\;\mathrm{dB}$, $\gamma_{\mathrm{com}}=-5\;\mathrm{dB}$, $r_{\mathrm{th}}=5\;\mathrm{bpcu}$).

In order to assess the effectiveness of our proposed LPI-based optimal power allocation strategy, Figure 6 shows a histogram of the comparisons of transmit power for radar waveform and communication rate employing different methods, which is conducted through $10^{4}$ Monte-Carlo trials. The results imply that the LPI-based optimal power allocation strategy outperforms the average power allocation method and the algorithm proposed by Chalise, B. K. in [1], in terms of the transmission power consumption for radar waveform and communication rate. Specifically, the LPI-based optimal power allocation strategy enables us to reduce the transmission power for radar waveform to 42.4% and 22.9% of that obtained by the average power allocation and the proposed algorithm in [1], respectively. On the other hand, both the LPI-based power allocation scheme and the algorithm proposed in [1] can reduce the transmission power for communication rate to 19.6% of that obtained by the average power allocation approach. Furthermore, the comparisons of probability of detection and communication rate utilizing different algorithms are given in Table 1, from which we can find that the presented strategy can guarantee the predefined thresholds of the probability of detection and communication rate. Therefore, we can conclude from the previous results that the total transmitted power for the joint system can be minimized by employing the LPI-based optimal power allocation strategy while guaranteeing a specified information rate for the communication receiver and desired target detection performance for the radar receiver.

## 5. Conclusions

In this paper, we have studied the LPI-based power allocation strategy for a joint bistatic radar and communication system. The analytically closed-form expression for the probability of false alarm is calculated, whereas the closed-form expression for the probability of detection is approximated by assuming that the SNR corresponding to the target surveillance path is much weaker than that corresponding to the line of sight path at the radar receiver. The basis of this algorithm is to exploit the optimization technique to minimize the total transmitted power for both information signal and radar waveform while guaranteeing a specified information rate for the communication receiver and the desired probabilities of detection and false alarm for the radar receiver. Then, the resulting optimization problem is solved through the well-known bisection search method. The LPI performance of the proposed strategy is evaluated through modeling and simulation and its superiority compared to other methods is illustrated. In future work, we will extend the system model and derivations to the MIMO system case (consisting of $M_{T}$ dedicated transmitters, $M_{\mathrm{rad}}$ radar receivers and $M_{\mathrm{com}}$ communication receivers), which is equivalent to the case with $M_{T}M_{\mathrm{rad}}$ monostatic radars and $M_{T}M_{\mathrm{com}}$ communication links.

## Figures and Tables

**Table 1:** Comparisons of probability of detection and communication rate employing different algorithms ($P_{\mathrm{Total}}=1000\;W$, $\delta_{D}=0.9$, $\delta_{\mathrm{FA}}=10^{-6}$, $\gamma_{\mathrm{rad}}=-10\;\mathrm{dB}$, $\gamma_{\mathrm{com}}=-5\;\mathrm{dB}$, $r_{\mathrm{th}}=5\;\mathrm{bpcu}$).

Methods	Probability of Detection	Communication Rate (bpcu)
LPIbasedoptimalpowerallocation	0.9	5
Averagepowerallocation	1.0	7.3139
ProposedalgorithmbyChaliseB.K. [1]	1.0	5
